# Sit-to-stand performance in children with cerebral palsy: a population-based cross-sectional study

**DOI:** 10.1186/s12891-024-07557-0

**Published:** 2024-06-11

**Authors:** Elinor Romin, Anna Lindgren, Elisabet Rodby-Bousquet, Erika Cloodt

**Affiliations:** 1 Habilitation Centre Child and Youth, Region Kronoberg, Växjö, Sweden; 2 Centre for Mathematical Sciences, Lund University, Lund, Sweden; 3 Department of Clinical Sciences Lund, Orthopaedics, Lund University, Lund, Sweden; 4 Centre for Clinical Research Västerås, Uppsala University-Region Västmanland, Västerås, Sweden; 5 Department of Research and Development, Region Kronoberg, Växjö, Sweden

**Keywords:** Cerebral palsy, Children, Sit-to-stand

## Abstract

**Background:**

Sit-to-stand (STS) is one of the most commonly performed functional movements in a child’s daily life that enables the child to perform functional activities such as independent transfer and to initiate walking and self-care. Children with cerebral palsy (CP) often have reduced STS ability. The aim of this study was to describe STS performance in a national based total population of children with CP and its association with age, sex, Gross Motor Function Classification System (GMFCS) level, and CP subtype.

**Methods:**

This cross-sectional study included 4,250 children (2,503 boys, 1,747 girls) aged 1–18 years from the Swedish Cerebral Palsy Follow-Up Program (CPUP). STS performance was classified depending on the independence or need for support into “without support,” “with support,” or “unable.” “With support” included external support from, e.g., walls and furniture. Physical assistance from another person was classified as “unable” (dependent). Ordinal and binary logistic regression analyses were used to identify associations between STS and age, GMFCS level, and CP subtype.

**Results:**

60% of the children performed STS without support, 14% performed STS with support, and 26% were unable or needed assistance from another person. STS performance was strongly associated with GMFCS level and differed with age and subtype (*p* < 0.001). For all GMFCS levels, STS performance was lowest at age 1–3 years. Most children with GMFCS level I (99%) or II (88%) performed STS without support at the age of 4–6 years. In children with GMFCS level III or IV, the prevalence of independent STS performance improved throughout childhood. CP subtype was not associated with STS performance across all GMFCS levels when adjusted for age.

**Conclusions:**

Independent STS performance in children with CP is associated with GMFCS level and age. Children with CP acquire STS ability later than their peers normally do. The proportion of children with independent STS performance increased throughout childhood, also for children with GMFCS level III or IV. These findings suggest the importance of maintaining a focus on STS performance within physiotherapy strategies and interventions for children with CP, including those with higher GMFCS level.

## Background

Sit-to-stand (STS) is one of the most commonly performed functional movements in a child’s daily life [1]. STS refers to the transitional movement toward an upright posture and involves shifting the body’s center of mass from a stable to a less stable position while extending the lower extremities [2]. STS is an important skill that allows the child to perform functional activities such as independent transfer and to initiate walking and self-care activities [3, 4]. STS is biomechanically demanding and challenges strength, stability, mobility, and coordination [5], and requires both dynamic and static postural control [6]. Normally, children perform STS independently by the age of 12–14 months [7, 8]. However, for children with cerebral palsy (CP), the impairment of the developing central nervous system leads to musculoskeletal dysfunction [9] and declined gross motor function [10], including challenges with STS performance that may affect their activities and self-care [11].

STS performance impacts the daily functioning of children with CP, and is a task in several motor assessments such as the Gross Motor Function Measure and theTimed Up and Go test as well as in training programs [12–15]. A previous review has indicated that STS training can enhance STS performance in individuals with disabilities [4]. Training aimed at improving STS in children with CP has been shown to be associated with improved self-care, and parents perceive the training as valuable and meaningful [16, 17]. Understanding how STS is performed can be useful for guiding rehabilitation interventions and providing insights to the therapist about the adaptations needed to facilitate the task. These interventions may involve adjustments such as altering seat height or implementing orthotic devices to improve stability and ease of execution [18–20]. STS performance is a predictor of independent walking ability [21], which highlights its clinical importance from an early age.

Previous studies to measure STS in children with CP are usually performed in a test situation or involves small groups of children, selected levels of the Gross Motor Function Classification System (GMFCS) or specific age groups [22–25]. There is a lack of prior research on STS performance in toddlers with CP to determine at what age they can accomplish the task and in adolescents to see whether STS performance is maintained, improved, or worsened during adolescence. The influence of age on STS performance has been analyzed only as an isolated factor and not combined with factors such as GMFCS level and subtype [26]. Therefore, the aim of this study was to describe STS performance in a national based total population of children with CP and its association with age, sex, GMFCS level, and CP subtype.

## Methods

This was a cross-sectional study based on register data from the Swedish Cerebral Palsy Follow-Up Program and registry (CPUP), which includes > 95% of all children with CP in Sweden, representing all 21 health care regions with a total population of 10.4 million inhabitants [27]. The study participants were all children with CP, aged 1 to 18 years, born between 2001 and 2022, who were reported into the registry between January 2020 and July 2023. Data from the most recent examination of all children were used. Children younger than 1 year of age and those with missing information about sex, subtype, or STS performance were excluded.

Children in the CPUP are followed with regular multiprofessional examinations at their local habilitation centre. These standardized examinations include assessment of gross and fine motor function, spasticity, mobility, and passive range of motion (https://cpup.se/in-english). The frequency of these examinations depends on the child’s GMFCS level and age, and varies from twice a year to every second year.

The child’s gross motor function was classified by their pediatric physiotherapist into levels I to V according to the expanded and revised version of the GMFCS [28]. The subtype was defined as the dominating neurological symptom of each child classified by their physiotherapist as spastic, dyskinetic, ataxic, or unclassifiable/mixed. The latter is used for example when both signs of dyskinesia and spasticity are equally prevalent, and a subtype cannot be delineated. Independent STS performance, from sitting on a chair to standing, was classified depending on the independence or need for support into “without support,” “with support,” or “unable.” “With support” included independent STS movement with external support from, for example, walls and furniture, but not from a person. Any physical assistance from another person was classified as “unable” (dependent). The classification of STS was based on knowledge of what the child does in everyday life (performance) and not their best capacity in a test situation. This study was designed in accordance with the STROBE guidelines for cohort studies.

### Statistical analysis

Age was categorized into six groups: 1–3, 4–6, 7–9, 10–12, 13–15, and 16–18 years. Pearson’s chi-squared test was used to identify differences in STS performance according to the variables age, sex, GMFCS level, and subtype. Regression analysis was used to analyze the explanatory factors (age and subtype) within each GMFCS level. Separate analyses were used for GMFCS level I–II, III and IV–V to count for the variation in STS performance. To analyze data for the small group of children with GMFCS level I or II who were unable to stand up without support, binary logistic regression was used with two levels of STS: "STS without support" compared with "STS with support + STS unable". In the group with GMFCS III, ordinal logistic regression was used in the analyses with all three levels of STS. To analyze data for the small group of children in GMFCS IV or V with STS ability, binary logistic regression was used with two levels of STS: “STS unable” compared with "STS with support + STS without suppor"t. Categorical variables are described by frequency (n) and percentage (%). Regression analyses are presented as odds ratios (ORs) with 95% confidence intervals and Bonferroni-adjusted *P* values. *P* values < 0.05 were considered to be significant. IBM SPSS Statistics (version 27; IBM Corp., Armonk, NY, USA) was used for the statistical analyses.

## Results

The original dataset included 4,390 children, and 4,250 remained after the exclusion of children younger than 1 year (*n* = 7) or lacking information about sex (*n* = 7), subtype (*n* = 38), or STS performance (*n* = 88). The excluded children were normally distributed and represented all GMFCS levels. Of the 4,250 children included, 2,503 were boys (59%) and 1,747 girls (41%). Their age range was 1 to 18 years, and their mean age was 10.3 years (SD 4.8). The distribution of age, sex, GMFCS level, and subtype are presented in Table 1.

**Table 1:** Sit-to-stand (STS) performance in relation to sex, age, GMFCS level, and subtype

			STS			
Variable	Level	Number of children n (%)	Unable n (%)	With support n (%)	Without support n (%)	*P* value*
**Sex**	Boy	2503 (59)	646 (26)	337 (14)	1520 (61)	**0.720**
	Girl	1747 (41)	451 (26)	250 (14)	1046 (60)	
**Age (years)**	1–3	415 (10)	126 (30)	106 (26)	183 (44)	**< 0.001**
	4–6	703 (17)	152 (22)	84 (12)	467 (66)	
	7–9	685 (16)	172 (25)	82 (12)	431 (63)	
	10–12	857 (20)	213 (25)	96 (11)	548 (64)	
	13–15	815 (19)	232 (29)	103 (13)	480 (59)	
	16–18	775 (18)	202 (26)	116 (15)	457 (59)	
**GMFCS level**	I	1969 (46)	3 (0)	44 (2)	1922 (98)	**< 0.001**
	II	684 (16)	11 (2)	92 (14)	581 (85)	
	III	348 (8)	34 (10)	254 (73)	60 (17)	
	IV	587 (14)	397 (68)	187 (32)	3 (1)	
	V	662 (16)	652 (99)	10 (2)	0 (0)	
**CP subtype**	Spastic	3456 (81)	675 (20)	471 (14)	2310 (67)	**< 0.001**
	Dyskinetic	485 (11)	334 (69)	63 (13)	88 (18)	
	Ataxic	149 (4)	14 (9)	31 (21)	104 (70)	
	Unclassifiable	160 (4)	74 (46)	22 (14)	64 (40)	
**Total**		**4250 (100)**	**1097 (26)**	**587 (14)**	**2566 (60)**	

Note: *Chi-squared test. The percentages presented have been rounded and the total may deviate from 100%

GMFCS, Gross Motor Function Classification System

**Fig. 1:**
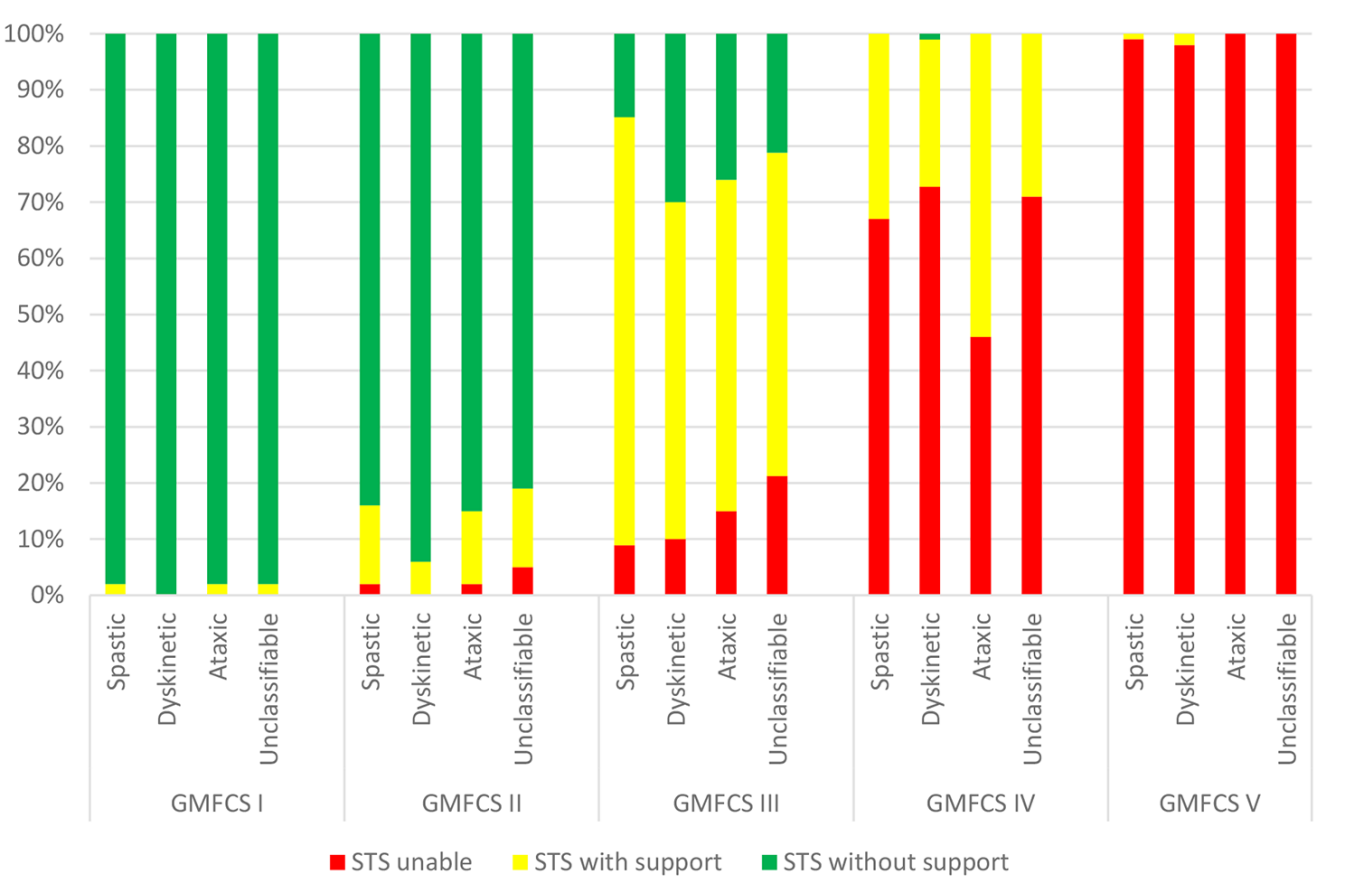
Sit-to-stand (STS) performance across CP subtypes within each GMFCS level

Almost three-quarters of the children with CP could perform STS independently: 60% performed STS without support and 14% with external support from walls or furniture. About one in four (26%) could not stand up or needed support from another person. The GMFCS level was strongly associated with STS performance. STS performance differed by age and subtype (Table 1). STS performance did not differ between CP subtypes within GMFCS levels I and V. By contrast, STS performance exhibited greater variability according to CP subtype within GMFCS levels II to IV (Fig. 1).

**Fig. 2:**
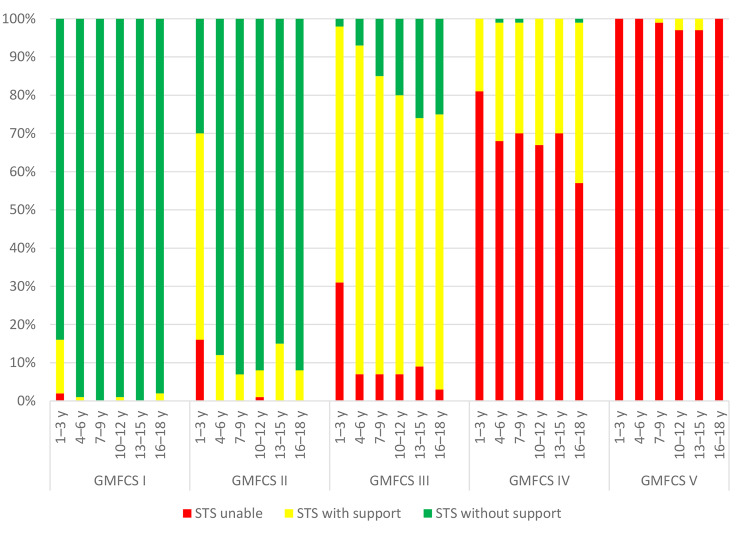
Sit-to-stand (STS) performance across age groups within each GMFCS level

In the group with GMFCS level III, the proportion of children with independent STS without support, performance increased incrementally with age. A similar trend was observed for the group with GMFCS level IV for independent STS with support. Children with GMFCS level I or II had consistent STS performance from age 4–6 years, and most could perform STS without support from that age (Fig. 2).

The CP subtype was not significantly associated with STS performance across all GMFCS levels when adjusted for age. However, in the analysis that included GMFCS level, subtype, and age simultaneously, only age 1–3 years was significantly associated with STS performance (Table 2).

**Table 2:** Logistic regression analyses of sit-to-stand performance and the association with age and subtype for GMFCS I–II, III, and IV–V analyzed separately

	GMFCS I or II^1^				GMFCS III^2^				GMFCS IV or V^1^		
	OR	95% CI	*P* value		OR	95% CI	*P* value		OR	95% CI	*P* value
**Age, years**											
1–3	20.00	10.00–33.33	**< 0.001**		11.48	4.75–27.72	**< 0.001**		2.91	1.42–5.98	**0.018**
4–6	1.12	0.56–2.33	1		3.04	1.23–7.51	0.079		1.53	0.88–2.65	0.67
7–9	0.47	0.20–1.08	0.37		1.83	0.82–4.09	0.716		1.64	0.94–2.87	0.42
10–12	0.69	0.33–1.43	1		1.50	0.69–3.29	1		1.31	0.78–2.20	1
13–15	1.10	0.55–2.22	1		1.19	0.57–2.50	1		1.43	0.86–2.39	0.84
16–18	Ref				Ref				Ref		
**Subtype**											
Dyskinetic	0.39	0.11–1.41	0.45		0.46	0.20–1.05	0.198		1.21	0.81–1.81	1
Ataxic	1.61	0.76–3.45	0.65		0.96	0.39–2.39	1		0.47	0.16–1.40	0.52
Unclassifiable	1.11	0.38–3.23	1		1.21	0.35–4.18	1		1.23	0.57–2.62	1
Spastic	Ref				Ref				Ref		
**GMFCS**											
I	Ref										
II	12.69	8.21–19.62	^3^								
IV									Ref		
V									31.34	16.34–60.13	^3^

Note: Data are presented as adjusted odds ratio (OR) with 95% confidence interval (CI) and Bonferroni adjusted *P* values. GMFCS, Gross Motor Function Classification System; Ref, reference

^1^ binary logistic regression, ^2^ordinal logistic regression, ^3^ the impact of GMFCS level on STS within the group

In the group with GMFCS I or II, nearly all children performed STS without support (98% and 85%, respectively) (Table 1). Among children with GMFCS I or II, toddlers aged 1–3 years were 20 times more unlikely (OR 20.0) to perform STS without support compared with older children. A child with GMFCS II was more unlikely (OR 12.7) to perform STS without support compared with a child with GMFCS I (Table 2).

In the group with GMFCS III, 17% performed STS without support, most of the children performed STS with support (73%), and 10% were unable to perform STS and needed additional support from a person (Table 1). Toddlers aged 1–3 years with GMFCS level III were more likely (OR 11.48) to be unable to perform STS independently (Table 2).

No children in the group with GMFCS IV or V performed STS without support. In the group with GMFCS level IV, 32% performed STS with support. In the group with GMFCS level V, 99% were unable to perform STS (Table 1). Toddlers with GMFCS IV or V were nearly three times more likely (OR 2.91) to be unable to perform STS even with support compared with the older age groups. Additionally, the risk was 31.3 times higher for a child with GMFCS V to be unable to perform STS, even with support, compared with a child with GMFCS IV (Table 2).

## Discussion

This study assessed STS performance in children with CP across all levels of motor function, CP subtypes, sex, and age groups. We found a significant association between STS performance in children with CP and their GMFCS level and with age. When considered within each GMFCS level, factors such as sex and CP subtype were not strongly associated with STS performance. The influence of GMFCS level on STS performance is expected given that STS is encompassed within the spectrum of gross motor functions assessed by the GMFCS.

We found that 60% of children performed STS without support. This percentage is similar to the 62% previously reported for 3–18-year-olds with CP [25]. However, we found a higher percentage of children unable to perform STS (26%) compared with the 18% reported by Rodby-Bousquet [25]. This disparity likely stems from our study’s inclusion of children of all ages from 1 to 18 years because many of the younger children had not yet acquired STS ability. However, the inclusion of all age groups in our study allowed us to show that children with CP learn STS later than their peers usually do.

We found that different CP subtypes had only a minor impact on STS within each GMFCS level. This aligns closely with the conclusions drawn by Gorter et al. in 2004 [29] and Rosenbaum et al. in 2010 [30], which highlight the limited predictive value of CP subtype concerning motor function compared with the GMFCS. In our study, when we examined the separate influence of CP subtype, we found that the small group of individuals with ataxia had better STS performance, whereas the reverse was seen in children with dyskinetic CP, although this distinction was not apparent when adjusting for GMFCS level.

In our study, children with CP acquired STS ability later than children without CP, who usually perform STS independently by the age of 12–14 months [7, 8]. Those studies on children without CP has a limited number of participants, which makes it more difficult to draw a definitive conclusion. However, the results align with the general understanding that children with CP achieve developmental milestones, such as walking, later than those without CP [31]. In contrast to expectations [32], we also found an association between increasing age and higher prevalence of individuals able to perform STS independently. Other research has shown that individuals with a higher GMFCS level may experience a decline in motor function as they age possibly because of deformities, pain, fatigue and reduced mobility [33, 34]. In our study, STS performance improved throughout childhood and adolescence even for those with more limited motor abilities (GMFCS level III or IV). The cross-sectional design of our study may account for the discrepancies from findings in other longitudinal studies showing a decreased gross motor function in adolescents with CP [32, 35].

The STS reflects the child’s performance in their everyday environment, contrasting with motor capacity, which means what one can achieve in a controlled setting. While children with CP show a correlation between motor performance and capacity, the former is notably influenced by environmental factors. Variables such as the level of assistance in daily activities, motivation, and social factors can significantly impact performance [36]. Furthermore, the ability to execute a task may vary across different environmental contexts [37]. Prior research has shown that families perceive reduced STS ability as a significant factor affecting their child’s independence and that this impacts parents both physically and emotionally [11]. Chaovalit et al. [16] highlighted the perceived value of STS training for families of children with a higher GMFCS level by providing a sense of hope that the children could continue to develop their physical abilities with further training and that training of STS can improve self-care and mobility for children with GMFCS level III or IV [17]. To enhance motor activity performance, it should be integrated into the child’s natural context, which is especially crucial for children at higher GMFCS levels [38]. STS training in combination with contextual and environmental adjustments can be important for children at higher GMFCS levels and the results of our study confirm that the proportion of children with independent STS performance increased incrementally with age in GMFCS level III–IV.

When discussing STS performance in children with CP, it is crucial to acknowledge the multifaceted nature of this challenging task [39]. Children with CP face added complexities when performing STS because of potential cognitive impairment [39], visual limitations [40], poor strength [24], reduced trunk control [41], and altered biomechanics [42]. It is equally vital to ensure that interventions do not concentrate solely on the physical aspects but also integrate elements that align with the child’s and family’s needs by focusing on both activity, participation, social factors and environmental factors [36, 43].

The study is conducted in a country that benefits from systematically following children with CP over an extended period and all children with CP receive regular interventions free of charge. This study has several limitations. First, its cross-sectional design restricts the ability to infer causation or temporal sequences between variables because it captured data at a single point in time and provides a snapshot rather than a longitudinal perspective. Second, reliance on registry data introduces the possibility of inaccuracies, potential errors, or missing information, which could impact the completeness and accuracy of the study’s outcomes. However, only 3.2% of children were excluded because of missing data or age < 1 year. Using registry-based data also provided a comprehensive coverage of more than 95% of all children with CP in Sweden. Moreover, these measurements adhere to a standardized protocol, which ensured consistency in data collection and registration processes.

The distribution of GMFCS levels may differ slightly between countries, but this discrepancy is reduced with GMFCS levels I–II combined, and GMFCS levels IV–V combined, aligning with the approach taken in the regression analysis of this study [44]. The distribution of GMFCS levels also varies among the Nordic countries, but the overall pattern remains similar, with GMFCS level I being the largest group, followed by levels II, V, IV, and with III being the smallest group [45].

The physiotherapy form does not differ between unilateral and bilateral spastic CP and do not allow analyses of potential differences within the spastic CP group. Both children unable to perform STS and those who need physical assistance of another person, are classified as “unable”. Even though this difference is important to facilitate transfers our primary focus was to investigate the proportion of children with independent STS performance without assistance of another person.

Preserving STS ability is important and we recommend that STS performance should be monitored even for children at higher GMFCS levels. For a comprehensive understanding of STS performance and to identify potential areas requiring adjustments such as seating height, orthotics, footwear and training, more detailed clinical assessments are necessary.

It would be interesting for future studies to explore how adjustments of environmental factors to facilitate STS performance may enhance participation in everyday situations for children with CP and the effect of physiotherapy interventions on STS performance. Investigating STS performance in adults with CP would also be valuable by identifying whether this ability declines during adulthood despite the lack of observable deterioration during childhood.

## Conclusions

This study provides evidence of a robust association between independent STS performance and GMFCS level in children with CP. The results indicate that children with CP acquire STS ability later than children without CP, and that most children with GMFCS level I–II perform STS without support from the age of 4–6 years. Incrementally more children with GMFCS III–IV perform STS independently with increasing age. STS training and adjustments of environmental factors could potentially further increase the proportion of children who perform STS independently, also those with higher GMFCS levels.

## Data Availability

The dataset analyzed for this study is available in the CPUP registry. Permission to access data is granted by KVB Region Skåne after ethical approval. Requests to access the dataset should be directed to https://vardgivare.skane.se/kompetens-utveckling/forskning-inom-region-skane/utlamnande-av-patientdata-samradkvb/.

## Declarations

### Authors’ information

ER is a physiotherapist and MSc at the Habilitation Centre in Växjö, Sweden. AL is a statistician and Assistant professor in mathematics at Lund University, Sweden. ERB is a physiotherapist and Associate professor at the department of Clinical Sciences, Orthopaedics, Lund University and at the Centre for clinical Research, Uppsala University, Västerås, Sweden. EC is a physiotherapist and PhD at the Habilitation Centre in Växjö and at the Department of Research and Development, Region Kronoberg and the Department of orthopaedics, Lund University, Sweden.

## Abbreviations

CP: Cerebral palsy
GMFCS: Gross Motor Function Classification System
OR: Odds ratio
STS: Sit-to-stand
